# Next-Generation Sequencing in the Mycology Lab

**DOI:** 10.1007/s12281-016-0253-6

**Published:** 2016-03-16

**Authors:** Jan Zoll, Eveline Snelders, Paul E. Verweij, Willem J. G. Melchers

**Affiliations:** Department of Medical Microbiology, Radboud University Medical Centre, Box 9101, 6500 HB Nijmegen, The Netherlands; Institut Pasteur, Unité de Mycologie Moléculaire, Paris, France

**Keywords:** Mycobiome, Next-generation sequencing, Whole genome sequencing, Resistance, Clinical mycology lab issues, Review

## Abstract

New state-of-the-art techniques in sequencing offer valuable tools in both detection of mycobiota and in understanding of the molecular mechanisms of resistance against antifungal compounds and virulence. Introduction of new sequencing platform with enhanced capacity and a reduction in costs for sequence analysis provides a potential powerful tool in mycological diagnosis and research. In this review, we summarize the applications of next-generation sequencing techniques in mycology.

## Introduction

Besides the human cells, the human body is comprised by microbial communities, the human microbiome, that plays important roles in a diversity of physiological processes. Many of these microbiota cannot be cultured in vitro and until the use of large-scale culture-independent molecular biological methods, the composition of the microbiome remained obscure. Recently, the Human Microbiome Project (HMP) and the Metagenomics of the Human Intestinal Tract (MetaHit) project were started [1, 2]. In these projects, the microbial composition of different mucosa, like the nasopharyngeal and oral cavities, the respiratory tract, skin, the gastrointestinal tract, and the urogenital tract are characterized. The majority of the studies were mainly focused on the bacterial microbiomes, but an increasing number of studies are published on the exploration of the virome and mycobiome. In the last few years, several research papers as well as excellent review papers are published on the composition of the human microbiomes in health and disease [3–6, 7•, 8••].

In the last few years, ongoing research gained more insight into the composition of the mycobiome in different sites of the human body [9–12]. Although the contribution of fungi is limited to approximately 0.1 % of the total microbiome, it is thought that fungi play a pivotal role in maintaining microbial communities and physiological processes in the body [6, 13, 14]. Host-microbiome interactions are responsible for development of several disorders. It becomes more clear that microbiota, including fungi, are in balance with each other and the host and that disturbance of this fine-tuned host-microbiome balance can be involved in disease [15]. Determination of the mycobiome in conjunction with the other microorganisms and host genetic background might gain insight into the biology in disease and the future development of personalized medicine.

In the last two decades, multiple new high throughput sequencing techniques were developed. Increasing sequencing capacity combined with a dramatic decrease in costs made these sequencing techniques available for microbiological labs for both research and diagnostic purposes [16]. Nowadays, a number of approaches are used in the sequence analysis of microorganisms. In deep sequencing, the entire DNA content of a biological sample is sequenced. For reliable results, a depth or coverage of 10 to 15 is required. This means that every single nucleotide in the sample is read at least 10 to 15 times. Due to the microbiological complexity of biological samples, an amplicon-based approach is used. Parts of the ribosomal RNA gene are amplified by PCR and subsequently sequenced. The microbiological composition can be reconstructed based on the ribosomal DNA (rDNA) sequences. Finally, in whole genome sequencing (WGS), a complete genome is sequenced from DNA isolated from a single microorganism. WGS can subsequently be applied in phylogenetic and drug resistance analyses.

The last few years, benchtop sequencing platforms became available like the Ion Torrent PGM and Illumina MiSeq. More recently, the Illumina NextSeq sequencer was introduced, combining a high capacity and relatively low costs. Approximately 30 to 40 fungal genomes can be sequenced in a single run on an Illumina NextSeq500 for approximately 100 euro per genome. Direct deep sequencing of clinical samples has already been proven as a diagnostic tool in viral infections [17, 18]. Elaboration of the whole genome sequencing technique for mycobiome determination in clinical samples seems to be next logical step.

## The Human Mycobiome

The microbiome consists of a complex mixture of microbiota (e.g. bacteria, fungi, and parasites). Although the microbiome has become synonymous for the collection of bacterial microbiota, the mycobiome consisting of resident mycobiota is an essential part of the microbiome.

The diversity of fungal species in the human body is still not fully understood. Over 390 species can be recognized within the human mycobiome [19]. Nevertheless, differences are observed between the microbiological composition of the respiratory- and the gastrointestinal tracts. These differences are thought to be reflections of the pH tolerance of the various fungal species and other environmental factors. Distribution of fungal species in the digestive tract was studied in detail by Gouba and colleagues [19]. In total, 335 species divided over 158 different genera were found in the digestive tract and the oral cavity of which 221 species were found only in the intestine and 88 species exclusively in the oral cavity. Remarkably, of the 247 species found in the digestive tract, only 59 were identified by in vitro culture and 207 by molecular techniques [19]. This indicates the substantial limitations in the detection of mycobiota by culture methods. In a study on the mycobiome composition of the oral cavity in 20 healthy individuals, 101 species were found by culture-independent methods [8••]. The species found belonged to 85 different genera of which 11 are considered nonculturable. A number of studies described the composition of the oral cavity mycobiome in healthy individuals and those with fungal disease. Ghannoum et al. screened oral rinses from 20 healthy individuals by sequence analysis of the ITS region of ribosomal genes [9]. On average, 15 fungal genera could be identified in which *Candida* and *Cladosporium* were most common. In addition other fungal genera associated with fungal disease were found including *Aspergillus*, *Cryptococcus*, *Fusarium*, and *Alternaria*. More than 50 % of the genera found by ITS sequencing are considered nonculturable. In another study, Dupuy et al. confirmed the results of Ghannoum [11]. However, they found *Malassezia* and *Epicoccum* as the most abundant genera in the oral mycobiome.

Changes in the mycobiome were found in patients with underlying diseases that compromise the host immunity. Several studies including HIV patients showed that the HIV infections correlate with increased abundance of *Candida*, *Aspergillus*, and *Fusarium* genera in the oral cavity [20•, 21]. Although the knowledge of the fungal composition of the long microbiome lags behind the extensive insights into the bacterial contents, it is known from several studies that the mycobiome of the lower respiratory tract is dramatically altered in cystic fibrosis and immunocompromised patients. Analysis of BAL samples and oral washes showed increased representation of medical relevant fungi as *Candida*, *Aspergillus*, and *Cryptococcus* in pulmonary diseases and immunocompromised patients [22, 23••]. During the last few years, several studies reported further insight into the composition of the mycobiome in various body sites in health and disease. A number of very informative review papers summarize the results of these studies [7•, 8••, 24••]. Nguyen et al. [7•] give an overview of the interactions between especially the lung mycobiome and different biomes in other body parts like the gut. The authors concluded that the “omics” approaches are essential to infer the emergent properties of polymicrobial communities. Understanding of fungal-bacterial interactions is essential in maintaining a healthy respiratory microbiome. Therefore, development of robust universal methodological strategies and implementation of these developments into large multicenter studies are required [7•]. The gut mycobiome and its relationships with intestinal diseases and inflammatory responses were reviewed by Mukerjee and colleagues. The authors stated that the fungal community is a critical player in gastrointestinal diseases. However, the links found between mycobiome composition in health and diseases reflect association rather than causation. For better understanding of the role of microbiota in health and disease, characterization of different classes of microorganisms like bacteria, fungi, and viruses is necessary [8••]. A comprehensive overview was given by Seed [24••] of mycobiome diversity between body sites and dynamics during human development, health, and disease.

Identification of microbiota has gained more speed by the introduction of metagenomics. Deep sequencing of clinical samples or microorganisms isolated from different body sites provides valuable information on the composition of the microbiome and the presence of potential pathogens. Moreover, advanced sequencing techniques enable a system biological approach of infectious diseases. The relationships between various microorganisms and between microorganisms and the human host can be studied and will gain more insight into pathogenesis and infection.

## Next-Generation Sequencing in Clinical Mycology

Next-generation sequencing (NGS) techniques have been applied in public health microbiology for outbreak monitoring and for metagenomic studies [25, 26]. The increasing performance of bench top sequencers such as the Illumina MiSeq and the Ion Torrent PGM is associated with an ongoing reduction in costs.

Translation of deep sequencing techniques into routine microbiological diagnostics on clinical samples seems a logical next step, thereby not only broadening the range of microorganisms that can be detected but also providing an additional characterization of the detected microbiota including mycobiota. Nevertheless, the abundance of fungal species in microbiota is relatively low. It was estimated that 0.1–1.0 % of the microbiota consists of fungal species [6, 13, 14]. To be successful in detecting fungi at the species or even subspecies level requires a capacity of at least 10^12^ to 10^14^ nucleotides per sequencing run per sample. This means that application of whole genome sequencing for the determination of mycobiomes for diagnostics in clinical samples is far beyond the possibilities delivered by currently available sequencers like Illumina NextSeq and HiSeq.

For these reasons, experimental data on mycobiomes are obtained by targeted sequencing of amplicons of the ITS region of the fungal ribosomal genes. Using sequencing platform like the Illumina NextSeq, complete microbiomic information can be extracted from clinical samples with amplicon sequencing of ITS regions or the entire ribosomal genes. Application of microbiomics in routine diagnostic mycology is thereby possible. Full characterization of the microbial community in different sites of the body can also give indications of the severity of fungal infection infections. Bittinger et al. [23••] demonstrated that relative abundances of bacteria and mycobiota in the lung can be used to distinguish between fungal colonization and fungal infection [23••].

## Next-Generation Sequencing in Mycology Research

Next-generation sequencing techniques are introduced in mycology research. Microsatellite analysis is the common approach to study the genetic diversity between fungal isolates. However, analysis of single nucleotide polymorphisms (SNPs) is a more accurate marker for the evaluation of recombination and genetic relationships [27]. Amplicon sequencing of the ribosomal ITS region and whole genome sequencing of fungal isolates can be used as the most discriminative approach in genetic research of various fungal pathogens like *Aspergillus* and *Candida* species and as a tool in taxonomic identification [27, 28]. Azoles play an important role in the management of fungal diseases, including itraconazole (ITC), voriconazole (VOR), posaconazole, and isavuconazole. The last two decades, an increase is reported of azole-resistant strains isolated from patients after prolonged azole treatment [29–36]. A number of common mechanisms of azole resistance can be recognized in fungi. First, the increased activity of efflux pumps results in a decrease in the intracellular drug concentration. Second, adaptation of target site of demethylases active in sterol biosynthetic pathways. Third, increase of azole target enzyme production. Mutations involved in these resistance mechanisms are found in a number of pathogenic fungi like *Aspergillus*, *Candida*, and *Cryptococcus* [29–36]. Garnaud and colleagues [34] analyzed 40 *Candida* isolates comprising the species *Candida albicans*, *Candida glabrata*, and *Candida parapsilosis*. Additionally, eight clinical antifungal resistant isolates and 23 sequential isolates collected from ten different patients were analyzed. Resistant isolates displayed several mutations in genes commonly involved in antifungal resistance including demethylases and efflux pumps [34]. Using forward and reverse genetic approaches, Ianiri and Idnurm [36] identified genes involved in various stages of the *Cryptococcus neoformans* life cycle. *Cryptococcus* homologs of 35 genes required for viability in ascomycetes were disrupted and evaluated for drug resistance, including genes involved in the ergosterol biosynthetic pathway [36].

The opportunistic fungus *Aspergillus fumigatus* is a pathogen causing a range of diseases in humans, including invasive aspergillosis in immunocompromised patients [37]. Additionally, azole-resistant *A. fumigatus* isolates have been obtained from the environment, where exposure to azole fungicides is considered to play a role [38, 39]. Since antifungal therapy is essential in the treatment of aspergillosis, more insight in mechanisms underlying the resistance phenotype is required. Systematic screening by whole genome sequencing of fungal strains isolated from patients with long-term azole treatment can obtain insight in mechanisms of resistance development by extensive use of azole or antifungal compounds in general.

The Cyp51A protein is a demethylase involved in the biosynthesis of ergosterol, a component of the fungal cellular membrane. Cyp51A is the main target of demethylase inhibitors like azoles. Several mutations are found in Cyp51A that are connected to fungal azole resistance. A variety of amino acid substitution in Cyp51A, including G54A, P216L, M220V, and Y121F are known to be responsible for azole-resistant phenotype. Additionally, duplication of 34 and 46 bp nucleotide stretches within the promoter region in the CYP51A gene were found responsible for an increase in CYP51A expression.

Cyp51A is a hotspot for mutations that confer azole resistance, but in an increasing number of resistant isolates the underlying mechanism remains unknown [40, 41]. In a study by Camps and colleagues, four isogenic *A. fumigatus* isolates were collected in which two isolates were azole resistant due to prolonged azole treatment [40]. Changes in treatment of chronically infected patients induce resistant *A. fumigatus* variants gaining more fitness that cannot be explained by the observed Cyp51A mutations. WGS of strains with an unexplained azole-resistant phenotype isolated from azole-treated patients with aspergillosis makes it possible to follow the development of genetic changes in the *Aspergillus* genome. In the study by Camps et al., whole genome sequence analysis of isogenic *A. fumigatus* isolates that had undergone an azole phenotype switch revealed several non-synonymous mutations. The challenge then is to correlate mutations with phenotypes. As several mutations had emerged in the isogenic isolates, sexual crossing experiments with selection of the progeny on azole resistance phenotype were required to show that azole resistance was associated with a P88L amino acid substitution in the CCAAT-binding transcription factor complex subunit HapE. Moreover, the HapE P88L mutation caused an increased CYP51A gene expression [40].

Fraczek and colleagues [41] compared twelve azole-resistant *A. fumigatus* isolates lacking Cyp51A mutations. They identified 20 putative azole transporter genes. Expression studies of the genes of interest, in the absence and presence of azoles, demonstrated in one isolate an increase in CDR1B efflux transporter gene expression by ITC. In another isolate, the CYP51A expression was increased 500 times in the presence of ITC. Although the mechanism of the azole resistance was not identified, the authors concluded that the cdr1B efflux transporter might be associated with Cyp51A independent ITC resistance. A whole genome comparison study of isogenic *A. fumigatus* isolates from patients with aspergillosis revealed various amino acid changes [42]. In one out of five isolates, a P216L amino acid substitution was found in Cyp51A in conjunction with several other non-synonymous mutations and deletions of clusters of genes. The other azole resistant isolated did not harbor cyp51A mutations.

Apart from whole genome sequencing, next-generation sequencing techniques offers more tools for the study of genetic changes due to environmental influences. Extracellular stimuli or stress factor as antibiotic compounds may induce modifications in gene expression profiles. Insight into early effects of antifungal compounds on fungal metabolism might help us learn more about molecular mechanisms involved in resistance development. Gene expression is regulated by epigenetic mechanisms like DNA methylation and hydroxylation. Early mechanisms in the development of resistance can be studied by epigenetic changes within the fungal genome. Cytosine methylation or hydroxylation modifies significantly the temporal expression of genes. Cytosine methylation for instance, is studied by the chemical conversion using bisulfate of cytosine into uracil. Cytosine modification rates can be found by comparison of the genomes with or without cytosine conversion.

Gene expression can be studied more directly by transcriptome analysis. Fungi are grown under various conditions where after the messenger RNAs (mRNAs) are extracted from the cells and sequenced after conversion into complementary DNA (cDNA). Comparison of the relative abundance of specific mRNAs isolated from fungi grown in the presence or absence of antifungal compounds will give insight into the early effects of these compounds on gene expression.

## Conclusions

Progress in new molecular tools and techniques might increase speed and sensitivity in the diagnosis of infectious fungal diseases, determination of mycobiomes, and in fungal research. Next-generation sequencing techniques will enhance the production of molecular data. Identification of mycobiota by culture-dependent methods is limited by the fact that the majority of fungal species cannot be cultured in vitro. Use of next-generation sequencing techniques is therefore a valuable extension in the diagnostic repertoire. However, capacity of sequencers is not unlimited. This means that application of whole genome sequencing for the determination of complex microbiomes for diagnostics in clinical samples is far beyond the possibilities delivered by currently available sequencers like Illumina NextSeq and HiSeq. The increase in capacity of sequencing platform and the ongoing decrease of sequencing costs, make microbiome analysis an attractive tool in mycology. Due to the extensive use of antifungal compounds, development of resistance in fungi has become a serious problem in human healthcare and agriculture. Whole genome sequencing and epigenetic analyses like transcriptomics and DNA methylation assay are powerful approaches in the study to molecular mechanism of resistance in fungi.

## References

[1] Peterson J, Garges S, Giovanni M, McInnes P, Wang L, The NIH HMP Working Group (2009). The NIH human microbiome project. Genome Res.

[2] Huttenhower C, Gevers D, Knight R, Abubucker S, Badger JH, Chinwalla AT (2012). Structure, function and diversity of the healthy human microbiome. Nature.

[3] Orgiazzi A, Bianciotto V, Bonfante P, Daghino S, Ghignone S, Lazzari A (2013). 454 Pyrosequencing analysis of fungal assemblages from geographically distant, disparate soils reveals spatial patterning and a core mycobiome. Diversity.

[4] Iliev ID, Funari VA, Taylor KD, Nguyen Q, Reyes CN, Strom SP (2012). Interactions between commensal fungi and the C-type lectin receptor Dectin-1 influence colitis. Science.

[5] Cui L, Morris A, Ghedin E (2013). The human mycobiome in health and disease. Genome Med.

[6] Huffnagle GB, Noverr MC (2013). The emerging world of the fungal microbiome. Trends Microbiol.

[7.•] Nguyen LDN, Viscogliosi E, Delhaes L (2015). The lung mycobiome: an emerging field of the human respiratory microbiome. Front Microbiol.

[8.••] Mukherjee PK, Sendid B, Hoareau G, Colombel J, Poulain D, Ghannoum MA (2015). Mycobiota in gastrointestinal diseases. Gastroenterol Hepatol.

[9] Ghannoum MA, Jurevic RJ, Mukherjee PK, Cui F, Sikaroodi M (2010). Characterization of the oral fungal microbiome (mycobiome) in healthy individuals. PLoS Pathog.

[10] Lu Q, van den Ende AH, de Hoog GS, Li R, Accoceberry J, Durand-Joly I (2011). Reverse line blot hybridisation screening of Pseudallescheria/Scedosporium species in patients with cystic fibrosis: RLBof Scedosporium spp.in cystic fibrosis. Mycoses.

[11] Dupuy AK, David MS, Li L, Heider TN, Peterson JD, Montano EA (2014). Redefining the human oral mycobiome with improved practices in amplicon-based taxonomy: discovery of Malassezia as a prominent commensal. PLoS ONE.

[12] Willger SD, Grim SL, Dolben EL, Shipunova A, Hampton TH, Morrison HG (2014). Characterization and quantification of the fungal microbiome in serial samples from individuals with cystic fibrosis. Microbiome.

[13] Rodríguez MM, Pérez D, Chaves FJ, Esteve E, Marin-Garcia P, Xifra G (2015). Obesity changes the human gut mycobiome. Sci Rep.

[14] Qin J, Li R, Raes J, Arumugam M, Burgdorf KS, Manichanh C (2010). A human gut microbial gene catalogue established by metagenomic sequencing. Nature.

[15] Kutikhin AG, Yuzhalin AE (2015). Editorial: recent discoveries in evolutionary and genomic microbiology. Front Microbiol.

[16] Caboche S, Audebert C, Hot D (2014). High-throughput sequencing, a versatile weapon to support genome-based diagnosis in infectious diseases: applications to clinical bacteriology. Pathogens.

[17] Zoll J, Rahamat-Langendoen J, Ahout I, de Jonge MI, Jans J, Huijnen MA (2015). Direct multiplexed whole genome sequencing of respiratory tract samples reveals full viral genomic information. J Clin Virol.

[18] Bavelaar HH, Rahamat-Langendoen J, Niesters HG, Zoll J, Melchers WJ (2015). Whole genome sequencing of fecal samples as a tool for the diagnosis and genetic characterization of norovirus. J Clin Virol.

[19] Gouba N, Drancourt M (2015). Digestive tract mycobiota: a source of infection. Med Mal Infect.

[20.•] Mukherjee PK, Chandra J, Retuerto M, Sikaroodi M, Brown RE, Jurevic R (2014). Oral mycobiome analysis of HIV-infected patients: identification of Pichia as an antagonist of opportunistic fungi. PLoS Pathog.

[21] Nasidze I, Li J, Quinque D, Tang K, Stoneking M (2009). Global diversity in the human salivary microbiome. Genome Res.

[22] Charlson ES, Diamond JM, Bittinger K, Fitzgerald AS, Yadav A, Haas AR (2012). Lung-enriched organisms and aberrant bacterial and fungal respiratory microbiota after lung transplant. Am J Respir Crit Care Med.

[23.••] Bittinger K, Charlson ES, Loy E, Shirley DJ, Haas AR, Laughlin A (2014). Improved characterization of medically relevant fungi in the human respiratory tract using next-generation sequencing. Genome Biol.

[24.••] Seed PC (2015). The human mycobiome. Cold Spring Harbor Perspectives in Medicine.

[25] Norman JM, Handley SA, Virgin HW (2014). Kingdom-agnostic metagenomics and the importance of complete characterization of enteric microbial communities. Gastroenterology.

[26] Underwood AP, Dallman T, Thomson NR, Williams M, Harker K, Perry N (2013). Public health value of next-generation DNA sequencing of enterohemorrhagic Escherichia coli isolates from an outbreak. J Clin Microbiol.

[27] Araujo R (2014). Towards the genotyping of fungi: methods, benefits and challenges. Curr Fungal Infect Rep.

[28] Dannemiller KC, Reeves D, Bibby K, Yamamoto N, Peccia J (2014). Fungal high-throughput taxonomic identification tool for use with next-generation sequencing (FHiTINGS). J Basic Microbiol.

[29] Bueid A, Howard SJ, Moore CB, Richardson MD, Harrison E (2010). Azole antifungal resistance in Aspergillus fumigatus: 2008 and 2009. J Antimicrob Chemother.

[30] Lockhart SR, Frade JP, Etienne KA, Pfaller MA, Diekema DJ (2011). Azole resistance in Aspergillus fumigatus isolates from the ARTEMIS global surveillance is primarily due to the TR/L98H mutation in the cyp51A gene. Antimicrob Agents Chemother.

[31] Snelders E, van der Lee HA, Kuijpers J, Rijs AJ, Varga J (2008). Emergence of azole resistance in Aspergillus fumigatus and spread of a single resistance mechanism. PLoS Med.

[32] Verweij PE, Howard SJ, Melchers WJ, Denning DW (2009). Azole-resistance in Aspergillus: proposed nomenclature and breakpoints. Drug Resist Updat.

[33] Howard SJ, Cerar D, Anderson MJ, Albarrag A, Fisher MC (2009). Frequency and evolution of azole resistance in Aspergillus fumigatus associated with treatment failure. Emerg Infect Dis.

[34] Garnaud C, Botterel F, Sertour N, Bougnoux M, Dannaoui E, Larrat S (2015). Next-generation sequencing offers new insights into the resistance of Candida spp. to echinocandins and azoles. J Antimicrob Chemother.

[35] Kanafani ZA, Perfect JR (2008). Resistance to antifungal agents: mechanisms and clinical impact. Clin Infect Dis.

[36] Ianiri G, Idnurm A (2015). Essential gene discovery in the basidiomycete Cryptococcus neoformans for antifungal drug target prioritization. mBio.

[37] Latgé JP (1999). Aspergillus fumigatus and aspergillosis. Clin Microbiol Rev.

[38] Verweij PE, Snelders E, Kema GH, Mellado E, Melchers WJ (2009). Azole resistance in Aspergillus fumigatus: a side-effect of environmental fungicide use?. Lancet Infect Dis.

[39] Snelders E, Huis In 't Veld RA, Rijs AJ, Kema GH, Melchers WJ, Verweij PE (2009). Possible environmental origin of resistance of Aspergillus fumigatus to medical triazoles. Appl Environ Microbiol.

[40] Camps SMT, Dutilh BE, Arendrup MC, Rijs AJMM, Snelders E, Huynen MA (2012). Discovery of a HapE mutation that causes azole resistance in Aspergillus fumigatus through whole genome sequencing and sexual crossing. PLoS ONE.

[41] Fraczek MG, Bromley M, Buied A, Moore CB, Rajendran R, Rautemaa R (2013). The cdr1B efflux transporter is associated with non-cyp51a-mediated itraconazole resistance in Aspergillus fumigatus. J Antimicrob Chemother.

[42] Hagiwara D, Takahashi H, Watanabe A, Takahashi-Nakaguchi A, Kawamoto S, Kamei K (2014). Whole-genome comparison of Aspergillus fumigatus strains serially isolated from patients with Aspergillosis. J Clin Microbiol.

